# Strengthening Ni-Coated CNT/Mg Composites by Optimizing the CNT Content

**DOI:** 10.3390/nano12244446

**Published:** 2022-12-14

**Authors:** Jilei Xu, Yizhuang Zhang, Zhiyuan Li, Yunpeng Ding, Xin Zhao, Xinfang Zhang, Hanying Wang, Changhong Liu, Xiaoqin Guo

**Affiliations:** 1 School of Materials, Zhengzhou University of Aeronautics, Zhengzhou 450046, China; 2 School of Materials Science and Engineering, Zhengzhou University, Zhengzhou 450046, China

**Keywords:** carbon nanotube, magnesium matrix nanocomposite, strengthening, strong interface bonding, mechanical property

## Abstract

The dispersion of carbon nanotubes (CNTs) is the bottleneck in CNT-reinforced metal matrix composites. In this work, CNT/Mg composites were prepared by grinding Mg powder and then dispersing CNTs via ball milling and hot pressing. The uniform distribution of Ni-coated CNTs in the matrix was achieved by optimizing the content of CNTs. Scanning electron microscope, high-resolution transmission electron microscopy and X-ray diffraction, optical microscopy, and compression tests were employed. With the CNT content being less than 1%, the CNTs can be evenly distributed in CNT/Mg composites, resulting in a sharp increase in strength. However, with the higher CNT content, the CNTs gradually cluster, leading decreased fracture strain and strength. Furthermore, the coated Ni in the CNTs reacts with the magnesium matrix and completely transforms into Mg_2_Ni, significantly enhancing the interface bonding. This strong interface bonding and the diffusely distributed Mg_2_Ni in the matrix significantly strengthen the CNT/Mg composite.

## 1. Introduction

As the lightest structural metal, magnesium alloy has many potential applications in the aerospace and transportation industries to reduce carbon emissions [1]. The low absolute strength and low stiffness are the bottlenecks of magnesium alloy, preventing its application. Thus, magnesium matrix composites are synthesized by adding reinforcement to the magnesium alloy matrix, achieving high strength and high stiffness [2].

Carbon nanotubes (CNTs) have a high aspect ratio, high tensile strength (about 60 GPa [3]), high thermal conductivity (about 3000 W/m/K [4]), excellent electrical properties, and low density [5]. As a result, CNTs have become some of the best reinforcements for magnesium matrices [6]. However, CNTs have large Van der Waals forces and easily cluster, making their dispersion in the matrix difficult [7]. Furthermore, CNTs have poor wettability to metal, leading to poor interface bonding. These two factors limit the improvement of the mechanical properties in CNT-reinforced magnesium matrix (CNT/Mg) composites and hinders their development and application.

In recent years, most methods to improve the dispersion uniformity of CNTs have mainly consisted of ball milling [8], liquid dispersion (ultrasonic treatment, mechanical stirring, surfactant addition, and so on), the in situ synthesis of CNTs [9], functionalization [10] (include surface plating), friction stir welding [11], stir casting [12], and 3D printing [13]. These methods have achieved good results. Among these methods, the ball milling method with inert gas protection is easy to operate and has low equipment requirements. Studies on strengthening the CNT/Mg interfaces have mainly included surface plating (Si [14], Ni [15], MgO [16], TiO_2_ [17], SiO_2_, SiC [18], etc.), CNT doping (boron doping, etc.), and the dispersion of CNTs in Al for the whole phase and followed by mixing with the Mg matrix [19]. Among these methods, the coating of metal layers on CNT surfaces is effective and simple [8]. With the enthusiasm for bionics [20], Xiang et al. [21] constructed CNT/Mg shell-like composites by electrophoretically depositing CNT layers on Mg foils and subsequent rolling them to optimize the distribution of CNTs and obtain better mechanical properties.

A previous study [8] by the same authors found that the grinding of the Mg powder and subsequent mixing via ball milling can make the CNTs more evenly distributed. However, the influence of the CNT content on the microstructure and mechanical properties of composites and the mechanism of the influence are still unclear. In addition, Ni-coated CNTs were used to improve the interfacial bonding, and the interface conditions need to be studied in depth. Hence, it is very necessary to study these topics. In this work, Ni-coated CNTs and pure magnesium powder were used to prepare CNT/Mg composites via the grinding of Mg powder, followed by mixing with CNTs and vacuum hot pressing. The objective of this study was to clarify the influence of the CNT content on the dispersion, interface, and mechanical properties of the composites and the mechanism of the influence, so as to contribute to the development of magnesium matrix composites.

## 2. Materials and Methods

**Figure 1:**
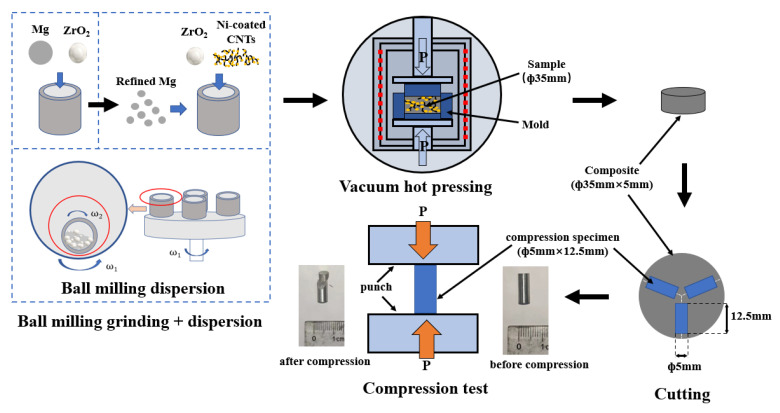
Preparation flow chart of the CNT/Mg composites.

The raw material used in the experiment were atomized magnesium powder (spherical diameter 20 μm; Yaotian new material Co., Ltd., Shanghai, China) and nickel-coated CNTs (outer diameter 30–50 nm, length < 10 μm, Ni content > 60 wt%, CNT content > 38 wt%; Jiacai Technology Co., Ltd., Chengdu, China). The preparation process of the CNT/Mg composite was divided into three steps—the grinding of the magnesium powder, the dispersion of CNTs by ball milling, and sintering by vacuum hot pressing, as shown in Figure 1. Most of the experimental operation processes, such as the powder loading, should be carried out in a glove box as far as possible to isolate the air and avoid the oxidation of the magnesium powder. In the process of magnesium powder grinding, the original magnesium powder and zirconia balls were put into sealed tanks and the ball milling was carried out for 60 h at a speed of 200 rpm in a planetary ball mill. In this process, a mass fraction of 5% stearic acid was added as the control agent, according to a rate of 1 wt.% per 12 h. The ball-to-material ratio was 10:1. In the process of dispersing the CNTs by ball milling, the ground magnesium powder, nickel-coated CNTs, and zirconia balls were added to the sealed tank under ball milling at 225 rpm for two hours. The ball-to-material ratio was the same as that of the grinding process. This composite powder was then placed in a graphite mold with an inner diameter of 35 mm. Cylindrical composites were prepared by sintering in a vacuum hot-pressing furnace (HP8-6-8GGD6A4A22, Centorr Vacuum Industries, Nashua, NH, USA). The sintering temperature was 550 °C and the holding time was 30 min. The air pressure was less than 5 Pa in the furnace. During the process of heating, the powder was heated to 400 °C at a rate of 10 °C per minute and stayed at this temperature for two hours, so that the stearic acid—the control agent mixed during ball milling process—was volatilized. Then, the powder was heated to 550 °C at the same rate and was held at this temperature for half an hour. Then, the sintered sample was cooled in the furnace.

A scanning electron microscope (SEM, JSM-7001F, JEOL, Tokyo, Japan) was used to observe the morphologies of the raw powder, ground powder, and mixed powder. The CNT/Mg powders were characterized using X-ray diffraction (XRD) techniques (Rigaku SmartLab, Tokyo, Japan). The composite was ground with silica sandpaper and polished with 1 micron diamond polishing paste. The sample was then corroded with a picric acid solution and the metallographic structure was observed using an optical microscope (BX51, Olympus, Tokyo, Japan). The sintered composite was cut into thin slices of 0.1 mm in thickness and ground to a thickness of 100 nm. Then the slices were cut into round slices with a diameter of 3 mm and ion thinning was carried out. Finally, these specimens were characterized via high-resolution transmission electron microscopy (HRTEM, TECNAI G2 F20S-TWIN, FEI company, Hillsboro, OR, USA). The composite was cut into strips measuring 12.5 mm × Φ 5 mm and the quasi-static compression performance was tested on the mechanical testing equipment (CMT5305, Suns, Shenzhen, China) at a compression speed of 0.0625 mm/min. Then, the stress–strain curves were obtained using the load and displacement data recorded during the compression process. The stress and strain in the compression test were calculated using the equations in the literature [22]. The GB/T 7314-2017 Chinese standard was used for the experimental tests. During the compression test, three specimens were used for each treatment level. The average value and standard deviation of the strength and fracture strain were calculated and analyzed in detail.

## 3. Results

### 3.1. Morphology

**Figure 2:**
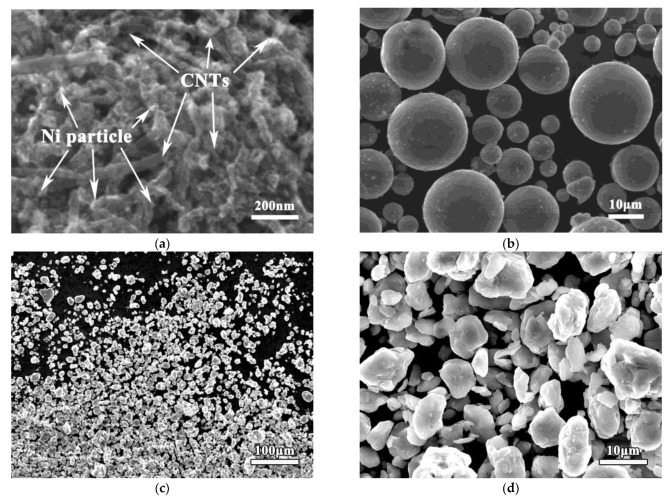
SEM morphology of as-received Ni-coated CNTs (**a**) and as-received (**b**) and ground (**c**,**d**) pure Mg powders.

Figure 2a shows the morphology of the as-received nickel-coated CNTs. After the chemical modification by nickel plating, there were many small Ni particles measuring less than 50 nm on the surfaces of the CNTs. This allowed the CNTs to bind more easily to the Mg matrix. Figure 2b shows the morphology of the as-received pure magnesium powder. The original atomized magnesium powder was a standard spherical shape. Its average diameter was about 20 μm. To reduce its size, the magnesium powder was ground by ball milling for 60 h. Its micromorphology after grinding is shown in Figure 2c,d. After a long period of grinding, the magnesium particles changed from a standard spherical to irregular lumpy shape. The particle size was also significantly reduced to about 3 μm. These particles were composed of many small broken magnesium particles welded together. This was due to the impact and friction [23] from ball milling for a long time, which meant the magnesium powder was periodically broken, inlaid, and welded. Due to the existence of the control agent during ball milling, the magnesium particles could not easily aggregate and grow, but mainly showed a tendency for grinding and refining.

**Figure 3:**
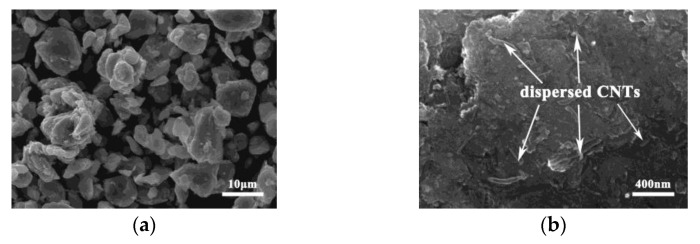

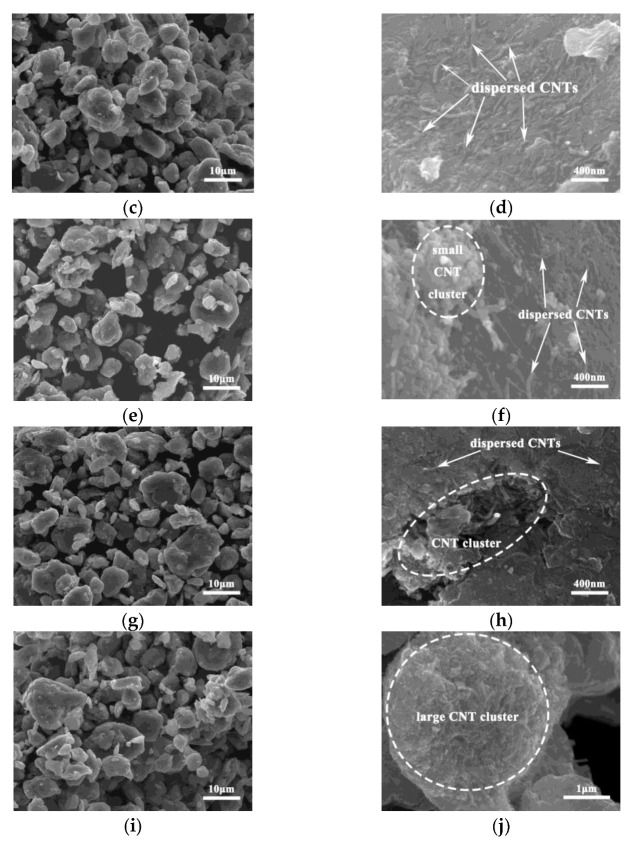
SEM morphology of CNT/Mg powders with different contents of CNTs: (**a**,**b**) 0.5 vol.%; (**c**,**d**) 1 vol.%; (**e**,**f**) 1.5 vol.%; (**g**,**h**) 2 vol.%; (**i**,**j**) 2.5 vol.%.

The ground magnesium powder was mixed with different amounts of nickel-coated CNTs by ball milling to prepare CNT/Mg powders with different contents of CNTs. The SEM image of the morphology is shown in Figure 3. The macromorphology of the Mg powder is similar. However, in the high-magnification diagram, the CNTs are evenly distributed on the surfaces of the magnesium particles when the CNT content is less than 1%. However, with the increase in the amount of CNTs, more and more CNTs cannot be dispersed evenly on the surfaces of the magnesium powder particles and the CNTs gradually agglomerate. When the CNT content reaches 2.0%, large clusters measuring 3 μm appear in the mixed CNT/Mg powder.

### 3.2. Microstructure and Phase

**Figure 4:**
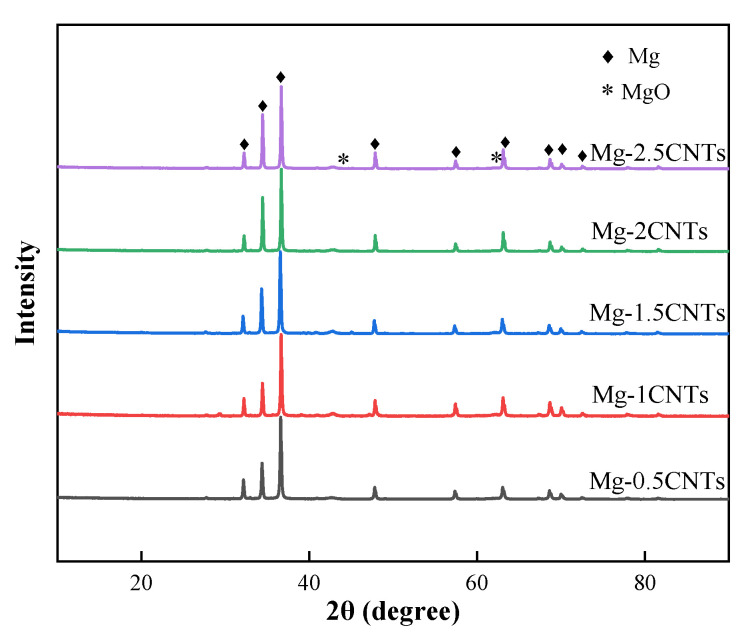
XRD patterns of sintered CNT/Mg composites with different contents of CNTs.

Figure 4 shows the X-ray diffraction patterns of CNT/Mg composites with different CNT contents. As can be seen, the composites are mainly composed of magnesium. In addition, the relatively low peak value of MgO proves the presence of a small amount of MgO in the composites. This may be caused by the contact and reaction between the Mg and O_2_ during powder preparation. Due to the small content of CNTs, no peaks of C and Ni phases can be found in the composites. Similar results also appeared in other literature reports [8].

**Figure 5:**
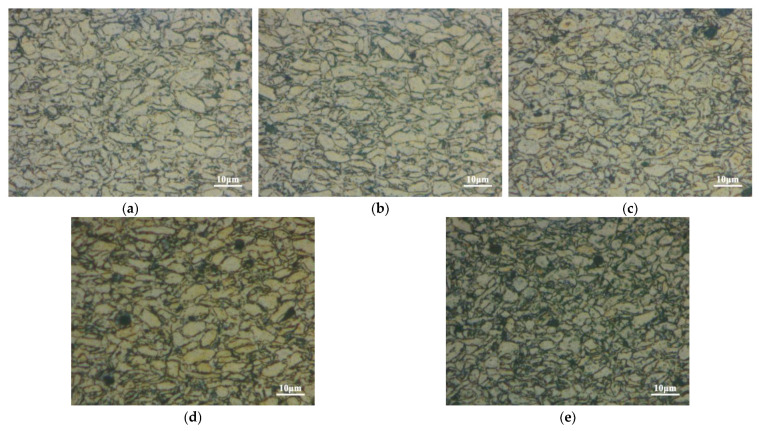
Microstructures of CNT/Mg composites with different contents of CNTs: (**a**) 0.5 vol.%; (**b**) 1 vol.%; (**c**) 1.5 vol.%; (**d**) 2 vol.%; (**e**) 2.5 vol.%.

Figure 5 shows the metallographic microstructure of the CNT/Mg composites with different CNT contents. The grain size and morphology of the matrix show little change with the increase in CNT content. However, when the CNT content is higher, there are more black spots in the microstructure. The black spots measure 2–4 μm in size in the 2.5% CNT/Mg composite. The size is similar to that of the CNT aggregates in mixed powders (Figure 3). Hence, it can be inferred that these black spots are CNT clusters.

**Figure 6:**
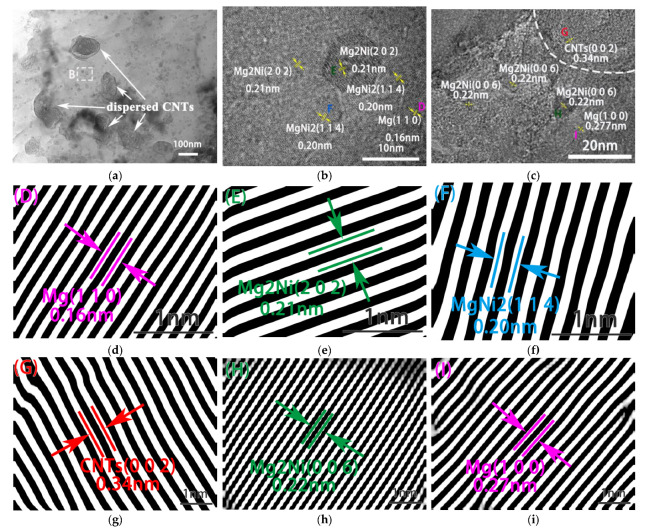
Low (**a**) and high (**b** and **c**) magnification TEM images of 1% CNT/Mg composites. The layer spacing of regions D, E, and F (**b**) are shown in (**d**–**f**). The layer spacing of regions G, H, and I (**c**) are shown in (**g**–**i**).

Figure 6 shows the TEM images of CNT/Mg composites with 1% CNTs. Figure 6a shows some separately distributed CNTs, proving that the CNTs are uniformly distributed in the matrix. Additionally, it can be seen that some small particles measuring about 10 nm are diffusely distributed in the matrix (in virtual frame B). This area is shown at high magnification in Figure 6b. An analysis of the atom-packed layer spacings shows that they are 0.2 nm and 0.21 nm, corresponding to the (114) and (202) planes of Mg_2_Ni, respectively. It was proven that a small number of Ni particles were separated from the surfaces of the CNTs and dispersed in the matrix under friction and impact during the ball milling process. In order to explore the bonding of CNT interfaces, the CNT/Mg interface is enlarged, as shown in Figure 6c. It can be seen from the analysis of atomic layer spacing that Mg_2_Ni intermediate alloy appears at the interface of the CNT and Mg matrix. However, there is no pure Ni phase at the interface. This result shows that the coated Ni reacted with the magnesium matrix and completely transformed into Mg_2_Ni during sintering.

### 3.3. Mechanical Property

**Figure 7:**
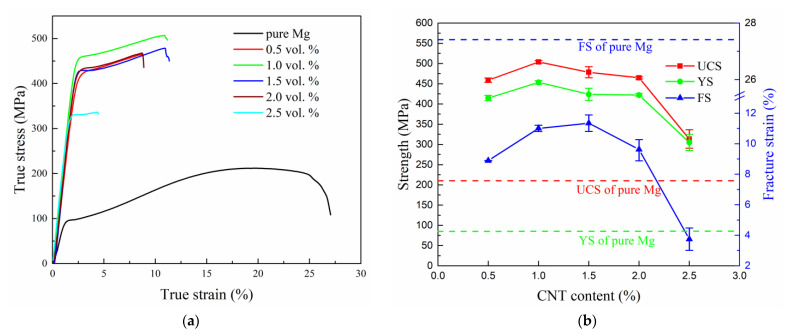
Effects of the CNT content on the compressive properties of CNT/Mg composites: (**a**) typical true stress–strain curve; (**b**) strength and fracture strain. The abbreviations of UCS, YS, and FS represent the ultimate compressive strength, yield strength, and fracture strain, respectively.

Figure 7 shows the true stress–strain curves and properties of CNT/Mg composites with different CNT contents under compression. Compared with pure Mg, the yield strength and ultimate compressive strength of the composites are greatly increased, while the fracture strain is decreased. When the CNT content is 1.0%, the yield strength and ultimate compressive strength of the composite are increased by 140% and 426% compared with pure Mg. The standard deviation of these data (Figure 7b) is very small, which means that the statistical variability is low.

Furthermore, with the increase in CNT content, the yield strength, ultimate compressive strength, and fracture strain of composites all firstly increase and then decrease. When the content of the CNTs is 1%, the yield strength and ultimate compressive strength of the composite are the highest, which are 453 MPa and 504 MPa, respectively. When the content of the CNTs is 1.5%, the fracture strain of the composite is maximal of 11.4%. When the content of the CNTs reaches 2.5%, the strength and fracture strain of the composite show a catastrophic decline. In conclusion, the composite has the best comprehensive performance when the CNT content is 1.0 vol.% under the range of research parameters.

### 3.4. Fracture Morphology

**Figure 8:**
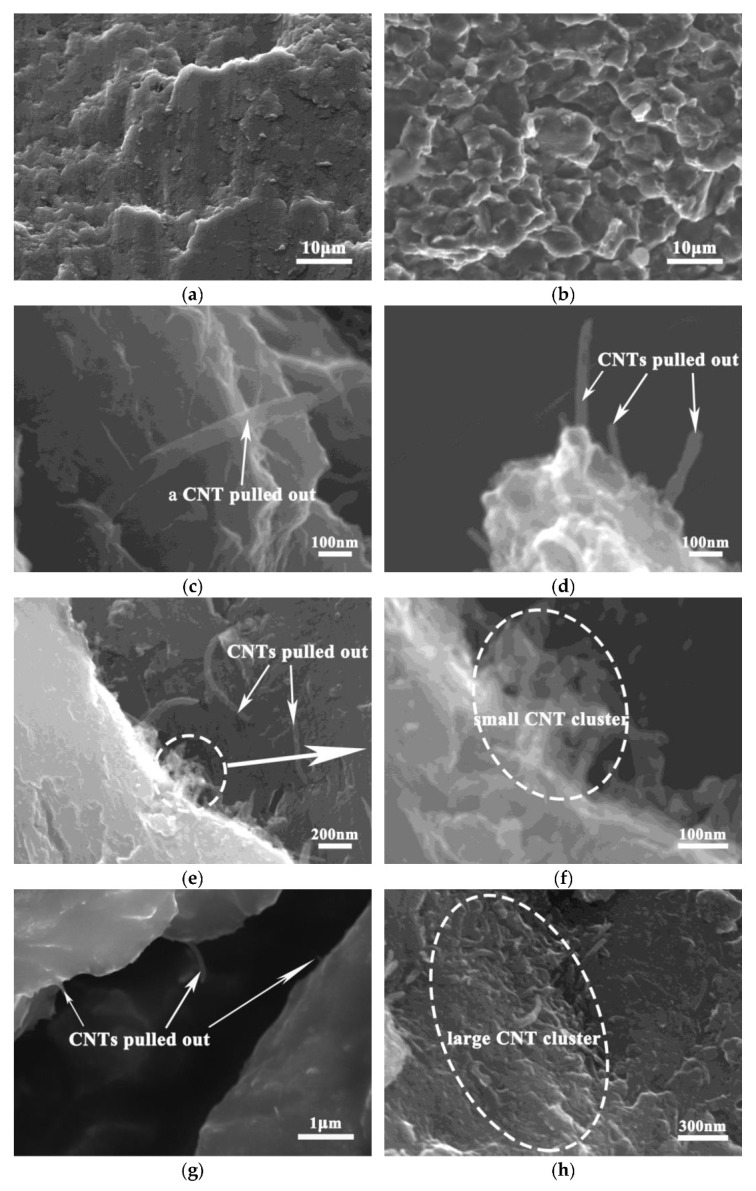
SEM images of the fracture morphologies in CNT/Mg composites with different contents of CNTs: (**a**) pure Mg; (**b**) CNT/Mg composites; (**c**) 0.5 vol.%; (**d**) 1 vol.%; (**e**,**f**) 1.5 vol.%; (**g**,**h**) 2.5 vol.%.

Figure 8 shows the fracture morphology of CNT/Mg composites with different contents of CNT. It can be observed in pure Mg (Figure 8a) that the fracture morphology is flat and platform-like, and no dimples are found. This is due to that the hexagonal close-packed structure of magnesium, which determines its poor plasticity and brittle, easily fractured nature. The fracture morphology of the composites is uneven, showing many granular bulges (Figure 8b). The dimensions of these bumps are consistent with those of the CNT/Mg particles. Therefore, this indicates that the crack propagates along the original particle boundary, which becomes the grain boundary after sintering and finally forms cracks. Compared with Figure 8c–h, it can be seen that when the CNT content is less than 1 vol.%, a large number of single CNT roots are pulled out at the fracture surface, showing a naked translucent shape. On the one hand, this shows that the CNTs have a uniform distribution, and on the other hand the CNTs play a bridging role in the deformation of the composites. With the increase in CNT content, more CNTs appears at the fracture and CNT clusters gradually begin to appear. CNT aggregates measuring about 2 μm in size can be observed at the fracture of the CNT/Mg composite with 2 vol.% of CNTs. This is consistent with the sizes of the black spots in the metallographic structures (Figure 5) and clusters in the mixed powder (Figure 3). The appearance of these clusters at the fracture surface indicates that they tend to cause stress concentration and become the source of cracks, eventually leading to cracking.

## 4. Discussion

After the grinding of magnesium powder, the collision and friction during ball milling lead to the grinding and periodic welding of the magnesium powder and the final magnesium particle size being significantly reduced [1] (Figure 2d). On the one hand, the decrease in particle size makes the surface area increase significantly. This provides a much larger space for the distribution of CNTs. On the other hand, the particle surfaces welded by small particles have a very high activity level, which makes it easier to combine them with the nickel layer of the CNTs (Figure 2a), meaning the CNTs can be uniformly dispersed on the surfaces of the magnesium particles (Figure 3 and Figure 6a).

The HRTEM results (Figure 6c) showed that the Ni coated on the CNT surfaces reacted with the magnesium matrix and completely transformed into Mg_2_Ni intermediate alloy at high temperatures during sintering. This interfacial reaction resulted in strong interfacial bond between the CNTs and magnesium matrix. This was conducive to the stress transfer between the CNTs and magnesium matrix during deformation, giving full play to the strengthening effect of the CNTs. In addition, Figure 6b proves that a small part of the coated Ni fell off from the surface of the CNTs during the ball milling process and reacted with the magnesium matrix to form the Mg_2_Ni phase with a particle size of only a few nanometers. These phases were evenly distributed in the magnesium matrix (Figure 6b) and played a role in dispersion strengthening. This dispersive phase works with the CNTs with strong interfacial bonding to significantly strengthen the CNT/Mg composites (Figure 7).

CNTs can achieve better dispersion when the CNT content is relatively small, as shown in Figure 3a–d. In the absence of clusters, the strong interfacial bonding between the CNTs and magnesium matrix enables the CNTs to play a role in load transfer (the bridging effect, as shown in Figure 8c–d) and dislocation blocking, thereby improving the bearing capacity of the Mg matrix. Therefore, both the strength and fracture strain of the composites increase with the increase in CNT content (Figure 7). However, when the CNT content exceeds 1.5%, the spaces on the surfaces of the magnesium powder particles are not enough to disperse these CNTs uniformly, so clusters are formed. After sintering, these clusters exist in the microstructures of the composites (Figure 5). When the CNTs are aggregated, they are unable to carry out load transfer [24] and cannot play a strengthening role during deformation. As a result, they become defects of the structure. On the other hand, their large size tends to cause stress concentration and they become the source of cracks, leading to premature cracking. Therefore, CNT aggregates occur at the fracture surfaces of composites with higher CNT contents (Figure 8f,h). Simultaneously, the strength and plasticity of the composites are reduced. The limitation of the preparation method used in this study is that the magnesium powder needs to be ground for a long time, which affects the preparation efficiency.

## 5. Conclusions

In this work, pure magnesium powder and nickel-coated CNTs were used as the raw materials. CNT/Mg composites were prepared via the grinding of Mg powder, dispersion of CNTs by ball milling, and hot-pressing sintering. The influence of the CNT content on the microstructure and mechanical properties of composites was studied. The main conclusions were as follows:

(1) When the CNT content is less than 1%, the CNTs can be evenly distributed in CNT/Mg composites, resulting in sharp increases in strength. However, with higher CNT content, the CNTs gradually agglomerate, leading to a decrease in the fracture strain and strength. The content of the CNTs has little influence on the grain size and morphology of the matrix;

(2) The coated Ni in the CNTs reacts with the magnesium matrix and completely transforms into Mg_2_Ni during sintering, which significantly enhances the interface. In addition, some of the Mg_2_Ni is diffusely distributed in the matrix, strengthening the CNT/Mg composite;

(3) The composite showed the most comprehensive performance when the CNT content was 1.0 vol.% under the range of research parameters. When the content of CNTs was 1%, the yield strength and ultimate compressive strength of the CNT/Mg composite were 453 MPa and 504 MPa, 140% and 426% higher than pure Mg, respectively.

This study proves that the Mg_2_Ni phase, generated at the CNT/Mg interface and dispersed in the matrix, has a great influence on the mechanical properties of the composites. The contents of CNTs in this study were different from those reported in some other studies, indicating that there are differences in the optimal CNT contents with different preparation methods.

## Data Availability

The data are available to download from https://pan.baidu.com/s/1K3pBjePFjRXRLkLL6rStFA?pwd=qfsf with the password “qfsf”, accessed on 14 November 2022.
